# Therapeutic Effects of Cold-Pressed Perilla Oil Mainly Consisting of Linolenic acid, Oleic Acid and Linoleic Acid on UV-Induced Photoaging in NHDF Cells and SKH-1 Hairless Mice

**DOI:** 10.3390/molecules25040989

**Published:** 2020-02-22

**Authors:** Hyeon Jun Choi, Bo Ram Song, Ji Eun Kim, Su Ji Bae, Yun Ju Choi, Su Jin Lee, Jeong Eun Gong, Hee Seob Lee, Chung Yeoul Lee, Bae-Hwan Kim, Dae Youn Hwang

**Affiliations:** 1 Department of Biomaterials Science, College of Natural Resources & Life Science/Life and Industry Convergence Research Institute, Pusan National University, Miryang 50463, Korea; rudwns546@naver.com (H.J.C.); 94sbr@naver.com (B.R.S.); prettyjiunx@naver.com (J.E.K.); suji130501@naver.com (S.J.B.); poiu335@naver.com (Y.J.C.); nuit4510@naver.com (S.J.L.); kos93589@naver.com (J.E.G.); 2 Department of Food Science and Nutrition, College of Human Ecology, Pusan National University, Busan 46241, Korea; heeseoblee@pusan.ac.kr; 3 Gangrim Organics, Miryang 50463, Korea; lcy9@hanmail.net; 4 Department of Public Health, Keimyung University, Daegu 42601, Korea; kim9399@kmu.ac.kr

**Keywords:** cold-pressed perilla oil, UV, photoaging, ROS, wrinkle formation, epidermis

## Abstract

Positive physiological benefits of several plant oils on the UV-induced photoaging have been reported in some cell lines and model mice, but perilla oil collected from the seeds of *Perilla frutescens L.* has not been investigated in this context. To study the therapeutic effects of cold-pressed perilla oil (CPO) on UV-induced photoaging in vitro and in vivo, UV-induced cellular damage and cutaneous photoaging were assessed in normal human dermal fibroblasts (NHDFs) and HR-1 hairless mice. CPO contained five major fatty acids including linolenic acid (64.11%), oleic acid (16.34%), linoleic acid (11.87%), palmitic acid (5.06%), and stearic acid (2.48%). UV-induced reductions in NHDF cell viability, ROS production, SOD activity, and G2/M cell cycle arrest were remarkably improved in UV + CPO treated NHDF cells as compared with UV + Vehicle treated controls. Also, UV-induced increases in MMP-1 protein and galactosidase levels were remarkably suppressed by CPO. In UV-radiated hairless mice, topical application of CPO inhibited an increase in wrinkle formation, transepidermal water loss (TEWL), erythema value, hydration and melanin index on dorsal skin of UVB-irradiated hairless mice. CPO was observed to similarly suppress UV-induced increases in epidermal thickness, mast cell numbers, and galactosidase and MMP-3 mRNA levels. These results suggest CPO has therapeutic potential in terms of protecting against skin photoaging by regulating skin morphology, histopathology and oxidative status.

## 1. Introduction

Photoaging is defined as premature skin aging due to continuous, long-term exposure to chronic UVA and UVB [1]. In photoaged skins, connective tissues are damaged and skins exhibited wrinkle formation, laxity, pigment condensation, and thickening [2]. UVB-induced reactive oxygen species (ROS) production in skin activates MAP kinase signaling pathways and consequently stimulates the secretions of inflammatory cytokines [3]. In addition, ROS overexpression increases the expressions of MMPs that can degrade collagen and elastic fibers [4]. Various plant oil and their mixtures have been investigated in terms of their abilities to suppress UV-induced photoaging in skin fibroblast cells and hairless mice. The mixture of primrose oils was found to inhibit UV-induced wrinkle formation via the regulations of MMP, MAP kinase, AP-1 transcription factor, and TGF-β2 [5], and the essential oils of *Cnidum officinale* makino and *Ligusticum chuanxiong* hort significantly inhibited UV-induced DNA damage and apoptosis in NIH 3T3 cells [6]. In addition, the topical application of almond oil reduced UV-induced histopathological alterations in mice skin [7], and patchouli and *Origanum hypericifolium* oils were reported to suppress UV-induced wrinkle formation, skin elasticity loss, and collagen and epidermal thickness increases in mice [8,9]. Furthermore, in hairless mice, *Angelica pubescens* oil improved the condition of UV-induced damaged skins [10]. However, the effects of perilla oil on UV-induced photoaging has not been previously investigated.

Perilla seeds and oil have attracted attention because they contain phytochemicals of therapeutic interest. Perilla oil contains fatty acids including linolenic acid (C18:3), linoleic acid (C18:2), palmitic acid (C16:1), oleic acid (C18:1) and stearic acid (C18:0) [11,12], and perilla seeds contain the phenolics rosmarinic acid, rosmarinic acid-3-O-glucoside, caffeic acid, ferulic acid, and caffeic acid-3-O-glucoside and the flavonoids apigenin, luteolin, and catechin [13]. In addition, perilla oil has been reported to have meaningful pharmacological effects in cancer, diabetes, asthma, microbial infectious diseases, inflammation, oxidative stress-related diseases, and cardiovascular disease [12,14], and perilla oil supplementation in diet was found to improve loperamide-induced constipation symptoms in albino rats and to enhanced cognitive function in elderly patients with mild cognitive impairment [15,16].

The present study was undertaken to investigate the anti-photoaging effects of cold-pressed perilla oil (CPO) in UV irradiated skin fibroblasts and an UV-induced mice model of photoaging and the mechanisms involved.

## 2. Results

### 2.1. Composition of CPO

**Figure 1:**
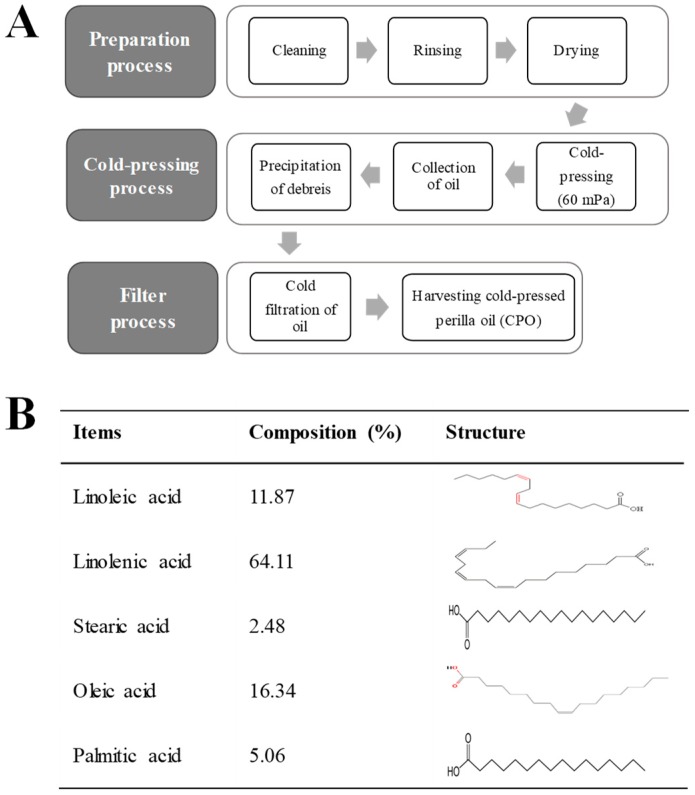
Preparation process and composition of CPO: (**A**) CPO was obtained from dried seeds of *P. frutescens* using a cold press at 60 Mpa and passing the crude oil through 6–8 μm membrane filters; (**B**) GC-MS showed CPO contained five fatty acids, that is, linoleic acid, linolenic acid, stearic aicd, oleic acid and palmitic acid.

As shown Figure 1B, CPO contained five major fatty acids, that is, linolenic, oleic, linoleic, palmitic, and stearic acids, which made up 64.11%, 16.34%, 11.87%, 5.06%, and 2.48% of CPO by volume, respectively, suggesting CPO is a good potential prospect for the treatment of UV-induced cell damage and photoaging.

### 2.2. Inhibitory Effect of CPO on UV-Induced NHDF Cell Death

**Figure 2:**
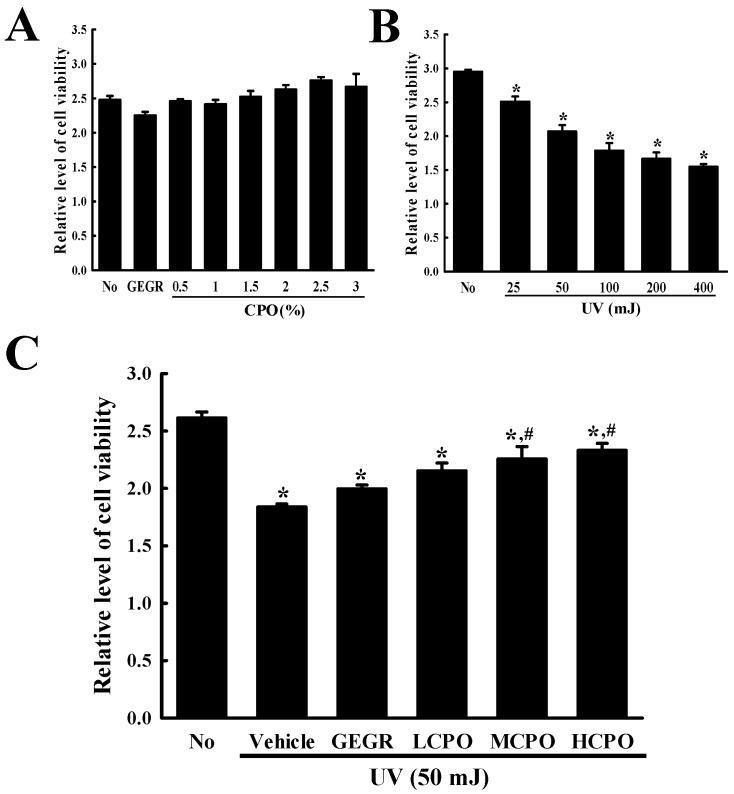
Viabilities of NHDF cells treated with UV and CPO. (**A**) Optimal CPO dose was determined using an MTT assay by treating cells with six different concentrations of CPO (0.5, 1, 1.5, 2, 2.5 and 3%). (**B**) Optimum UV dose was determined by exposing NHDF cells to UV at five levels (25, 50, 100, 200 and 400 mJ). (**C**) Viabilities of UV + CPO treated cells. After exposure 50 mJ of UV and treatment with different concentrations of CPO for 24 h, cell viability was measured using MTT assay. Two to three wells per group were MTT assayed and optical densities were measured in duplicate. Data are reported as the means ± SD. * *p* < 0.05 compared to the No group. # *p* < 0.05 compared to the UV + Vehicle group. Abbreviations: LCPO, low concentration CPO; MCPO, medium concentration CPO; HCPO, high concentration CPO; GEGR, gallotannin-enriched extract of Galla Rhois.

Initially, we measured the viabilities of cells treated with UV and CPO at different concentrations. Treatment with CPO at 0.5, 1, 1.5, 2, 2.5, or 3% for 24 h did not cause significant cell death although treatment with CPO at 1% to 2.5% show a tendency to slightly increase viability (Figure 2A). However, increasing UV radiation dose from 25 mJ to 400 mJ dose-dependently decreased cell viability (cell viability decreased by ~50% in cells exposed to 400 mJ) (Figure 2B). Based on above results, the optimal dosages of UV radiation and CPO were taken to be 50 mJ and 2.5%.

Next, we investigated the inhibitory effect of CPO on UV-induced cell death. To achieve this, we investigated cell viabilities after treating cells with 50 mJ of UV and CPO at 0.625%, 1.25% and 2.5%. Viabilities were lower in UV + Vehicle group than in treatment naïve controls, but dose-dependently higher in the UV + LCPO, UV + MCPO, and UV + HCPO groups (Figure 2C). These observations were in-line with morphological features (Data not shown). Accordingly, these results suggest that CPO treatment can prevent UV-induced NHDF cell death.

### 2.3. Inhibitory Effect of CPO on UV-Induced Reactive Oxygen Species (ROS) Production and Superoxide Dismutase (SOD) Activation in NHDF Cells

**Figure 3:**
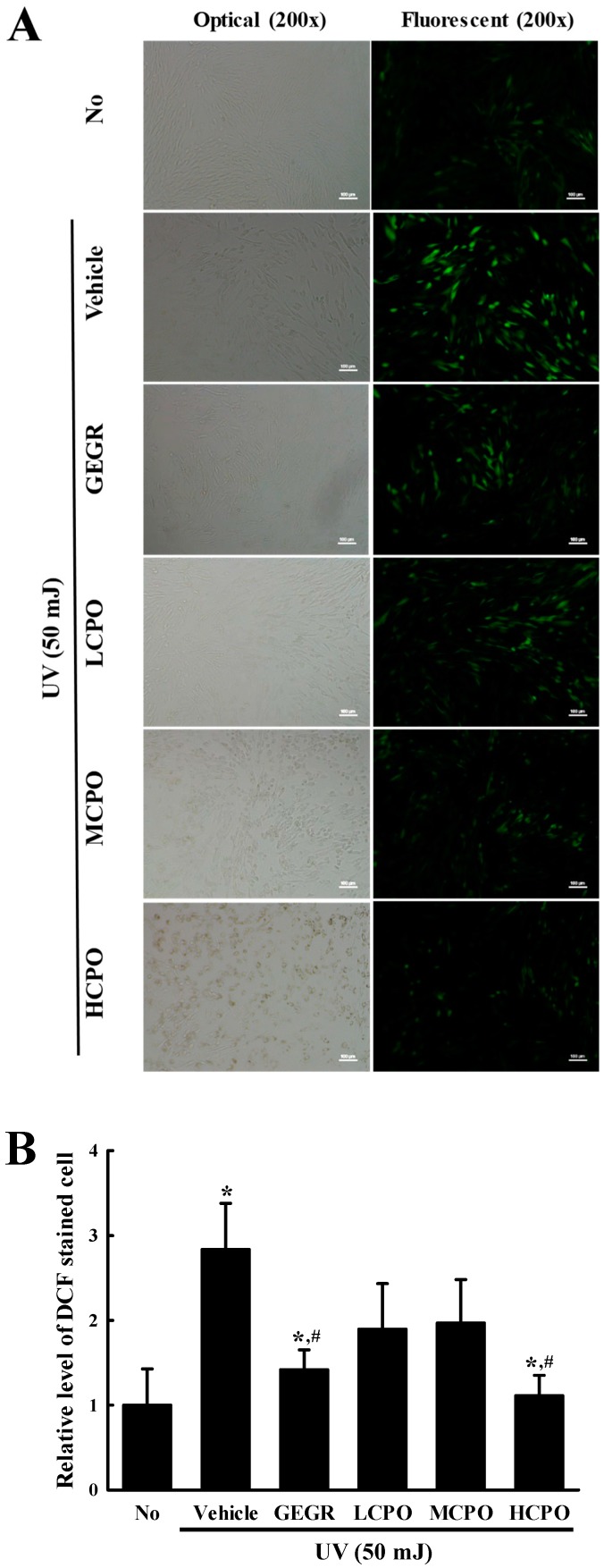
Determination of intracellular ROS production. (**A**) Image of green fluorescence in NHDF cells. After DCF-DA treatment, green fluorescence in cells was observed under a fluorescent microscope (Eclipse TX100, Nikon, Tokyo, Japan). NHDF cells were also examined under an optical microscope (left column) and a florescence microscope (right column) at 200× magnification. (**B**) Level of green fluorescence in NHDF cells. Signal intensities were quantified using ImageJ software [17]. Data are reported as the means ± SD. * *p* < 0.05 compared to the No group. # *p* < 0.05 compared to the UV + Vehicle group. Abbreviations: LCPO, low concentration CPO; MCPO, medium concentration CPO; HCPO, high concentration CPO; GEGR, gallotannin-enriched extract of Galla Rhois.

**Figure 4:**
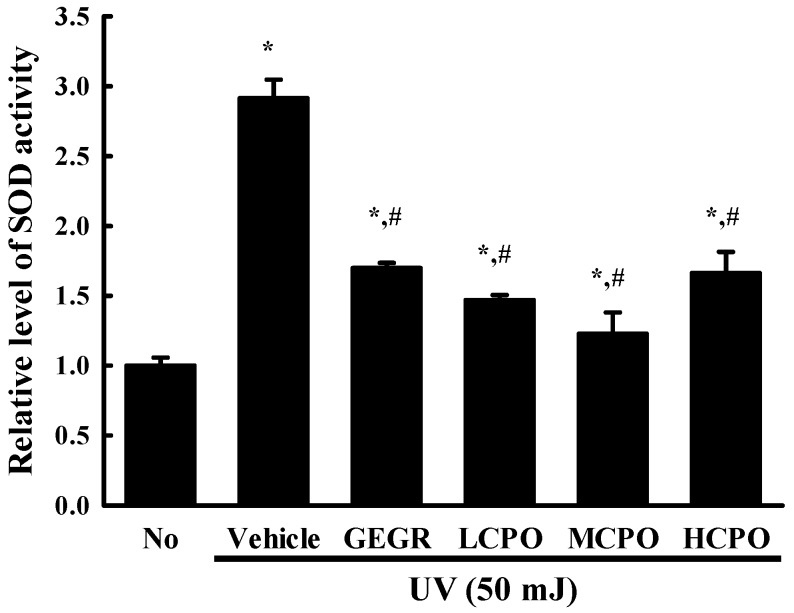
SOD activities in UV + CPO treated NHDF cells. SOD activities were measured in cell lysates as described in Materials and Methods. One SOD unit was defined as the amount of enzyme in 20 μL of sample solution that inhibited the reduction of WST-1 with superoxide anion by 50%. Two to three wells per group were used and SOD activity was measured in duplicate. Data are reported as the means ± SD. * *p* < 0.05 compared to the No group. # *p* < 0.05 compared to the UV + Vehicle group. Abbreviations: LCPO, low concentration CPO; MCPO, medium concentration CPO; HCPO, high concentration CPO; GEGR, gallotannin-enriched extract of Galla Rhois.

To investigate whether the inhibitory effect of CPO on UV-induced cell death was associated with its anti-oxidative effect in NHDF cells, ROS production and SOD activity were measured in UV + CPO treated cells. ROS production was remarkably increased in UV + Vehicle treated NHDF cells without any significant morphologic change. However, these levels were significantly and dose-dependently decreased in the UV + CPO treated groups. UV + HCPO treated group showed complete recovery to treatment naïve control levels (Figure 3). In addition, CPO had a similar inhibitory effects on SOD activity. Increased SOD activity in the UV + Vehicle group was significantly inhibited in the UV + CPO treated groups, although dose-dependency was not observed (Figure 4). These results indicate that the high anti-oxidant activity of CPO was probably associated with its inhibitory effects on UV-induced cell death.

### 2.4. Regulatory Effect of CPO on UV-Induced Cell Cycle Arrest in NHDF Cells

**Figure 5:**
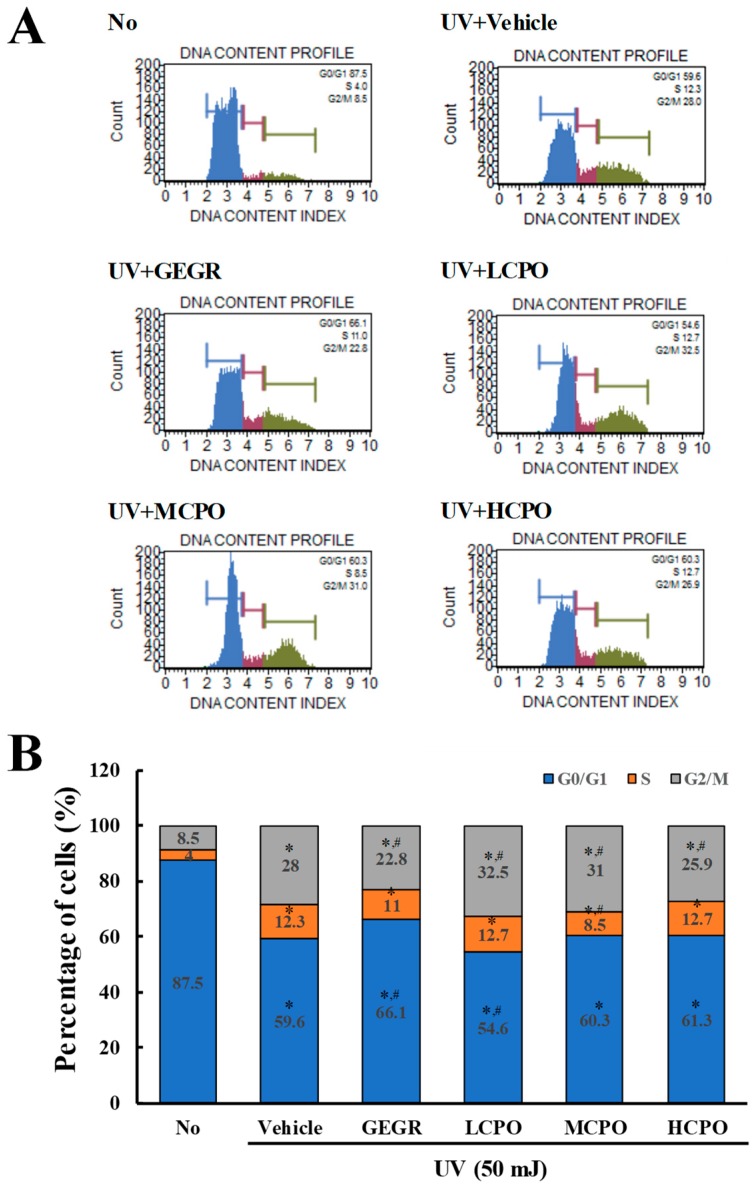
Cell cycle analysis after UV + CPO treatment. (**A**) Cell cycle histograms obtained using flow cytometry. Cell cycle distributions were determined by flow cytometric analysis of propidium iodide (PI) stained NHDF nuclei. (**B**) Percentage of cell numbers in each cell cycle. Numbers of cells in the G0/G1, S, and G2/M stages were determined after UV + CPO treatments. Two to three wells per group were PI stained and cell numbers in each phase were measured in duplicate. Data are reported as the means ± SD. * *p* < 0.05 compared to the No group. # *p* < 0.05 compared to the UV + Vehicle group. Abbreviations: LCPO, low concentration CPO; MCPO, medium concentration CPO; HCPO, high concentration CPO; GEGR, gallotannin-enriched extract of Galla rhois.

To investigate the regulatory effects of CPO on UV-induced cell cycle, cell cycle distributions were determined by FACS after treating cells with different CPO doses for 48 h. Numbers of cells in the G2/M and S phases were higher in UV + Vehicle treated group than in treatment naïve controls, whereas numbers in the G0/G1 phase were lower. Numbers in the G2/M phase were slightly lower after treatment with UV + GEGR and UV + HCPO, but numbers in the S phase were not (Figure 5).

### 2.5. Inhibitory Effect of CPO on UV-Induced MMP Expression and Galactosidase Level in NHDF Cells

**Figure 6:**
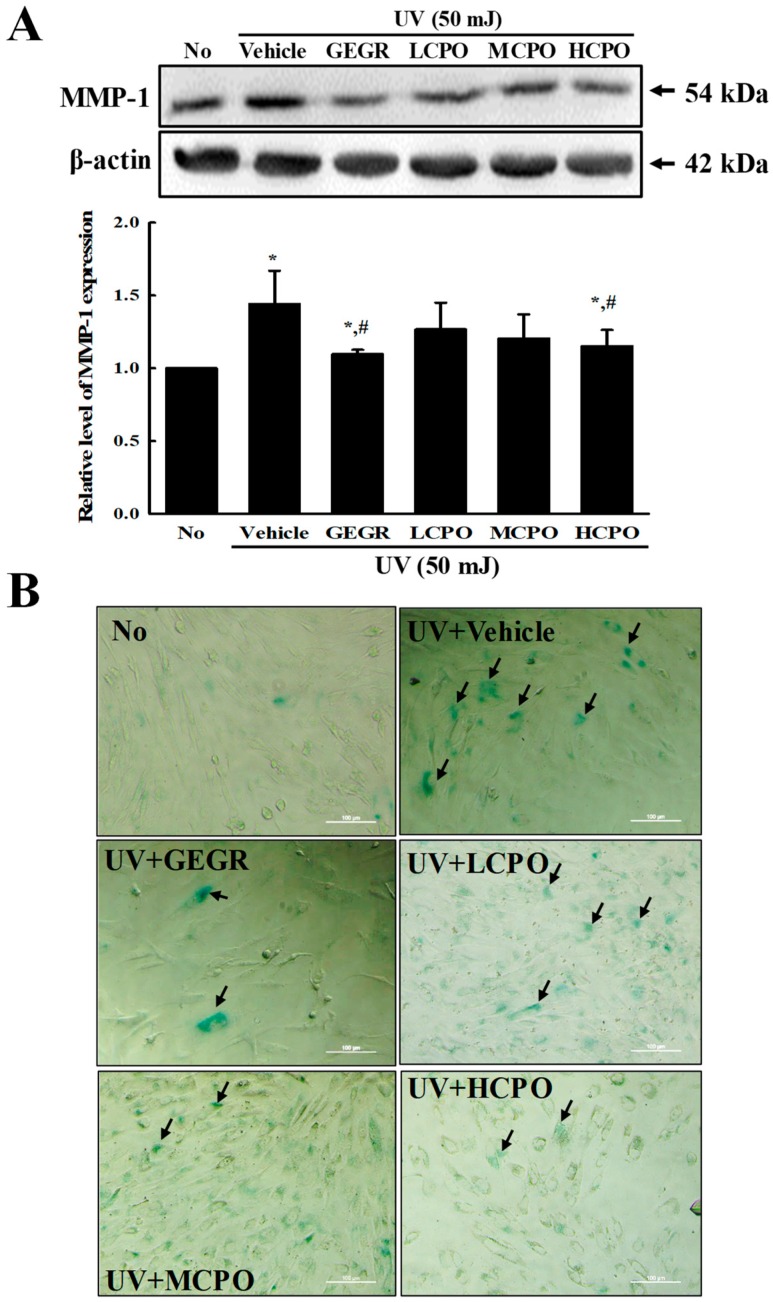
MMP-1 expression and galactosidase staining analyses of UV + CPO treated cells. (**A**) Western blot for MMP-1 protein. The level of MMP-1 and β-actin protein in lysates were determined by Western blotting using HRP-labeled anti-rabbit IgG antibody. Band intensities were measured using an imaging densitometer. The relative levels of MMP-1 protein were calculated versus β-actin protein. Two to three dishes per group were combined for western blotted analysis and samples were assayed in duplicate. (**B**) Galactosidase staining analyses. After incubation with galactosidase staining solution at 37 °C, color intensity in the cells were observed at 400× magnification under a microscope. Arrows indicated a X-gal stained cell. Two to three dishes per group were combined for western blotted analysis and samples were assayed in duplicate. Data are reported as the means ± SD. * *p* < 0.05 compared to the No group. # *p* < 0.05 compared to the UV + Vehicle group. Abbreviations: LCPO, low concentration CPO; MCPO, medium concentration CPO; HCPO, high concentration CPO; GEGR, gallotannin-enriched extract of Galla Rhois.

To examine the inhibitory effects of CPO on biomarkers of skin aging, we measured changes in the levels of MMP-1 and β-galactosidase in UV + CPO treated NHDF cells. β-galactosidase was regarded to be one of biomarkers for cellular senescence because this enzyme can hydrolysis β-galactosides into monosaccharides in only senescent cells [18]. MMP-1 expression and β-galactosidase staining degree were enhanced after UV radiation, but were both remarkably lower in the UV + CPO group (Figure 6). These results indicate that CPO inhibits the skin aging of UV-treated NHDF cells by suppressing the UV-induced upregulations of MMP-1 and β-galactosidase.

### 2.6. Protective Effects of CPO on UV-Induced Phenotypical and Histological Changes in the Skin of Hairless Mice

**Figure 7:**
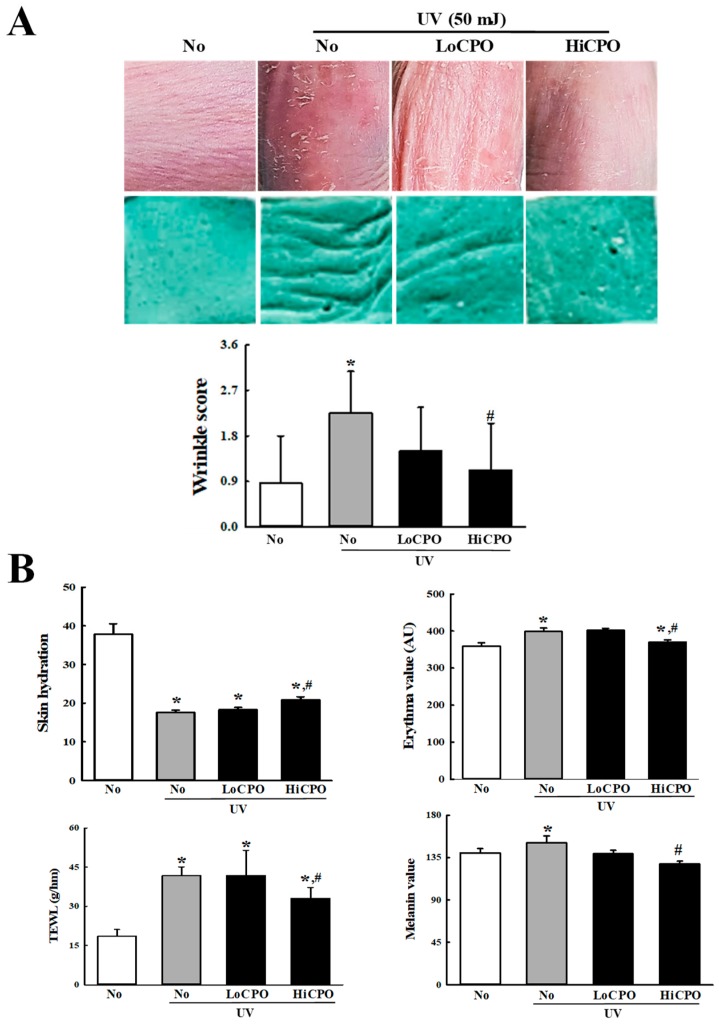
Skin phenotypical analyses of UV + CPO treated mice. (**A**) Wrinkle formation on the skin was measured by replica grading after 4 weeks of CPO treatment. Wrinkles were assessed as described by Bissett et al. [19] (grade 0, no wrinkles; grade 1, a few shallow wrinkles; grade 2, some wrinkles; grade 3, several deep wrinkles). (**B**) TEWL, skin hydration, and erythema and melanin indices of dorsal skins were analyzed in triplicate as described in Materials and Methods. Three to five mice per group were used and two different dorsal skin areas per mouse were assayed in duplicate. Data are reported as the means ± SD. * *p* < 0.05 compared to the No group. # *p* < 0.05 compared to the UV + No group. Abbreviations: LoCPO, low amount CPO; HiCPO, high amount CPO.

**Figure 8:**
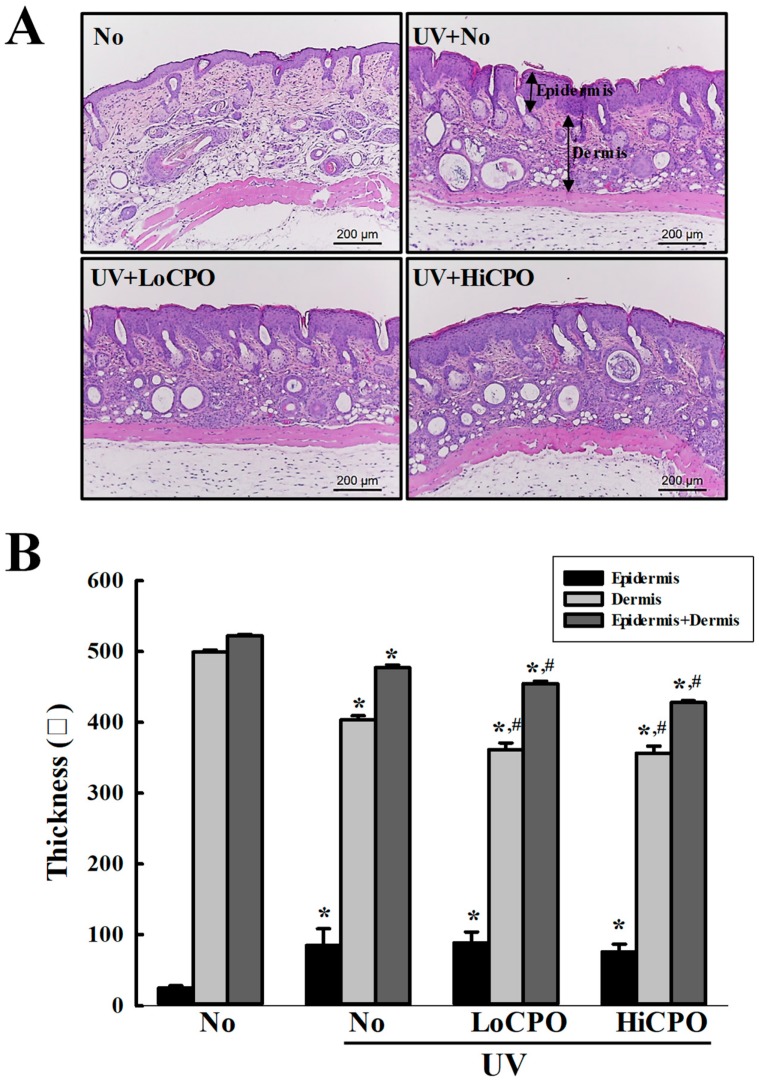
Histopathological structures of skins. (**A**) Image of H&E stained skin tissue. The dorsal skin tissues of hairless mice were fixed in 4% formaldehyde and stained with H&E solution. Histological changes were observed at 200× magnification under a microscope. (**B**) Epidermal and dermal thickness. H&E stained slides were prepared for three to five mice per group and epidermal thicknesses were assayed in duplicate for each mouse. Data are reported as the means ± SD. * *p* < 0.05 compared to the No group. # *p* < 0.05 compared to the UV + No group. Abbreviations: LoCPO, low amount CPO; HiCPO, high amount CPO.

Wrinkle scores, which included consideration of wrinkle depths and numbers, were remarkably increased by UV exposure, but CPO treatment significantly reduced these increases (Figure 7A). In addition, CPO treatment also significantly reduced UV-induced changes in transepidermal water loss (TEWL), skin hydration, and erythema and melanin indices although UV exposure increased TEWL, and erythema and melanin indices, but decreased skin hydration. Furthermore, these values of the UV + HiCPO group were similar to those of No group (Figure 7B). In addition, we also found CPO effectively prevented UV-induced skin thickening (Figure 8).

### 2.7. Inhibitory Effects of CPO on UV-Induced Skin Aging

**Figure 9:**
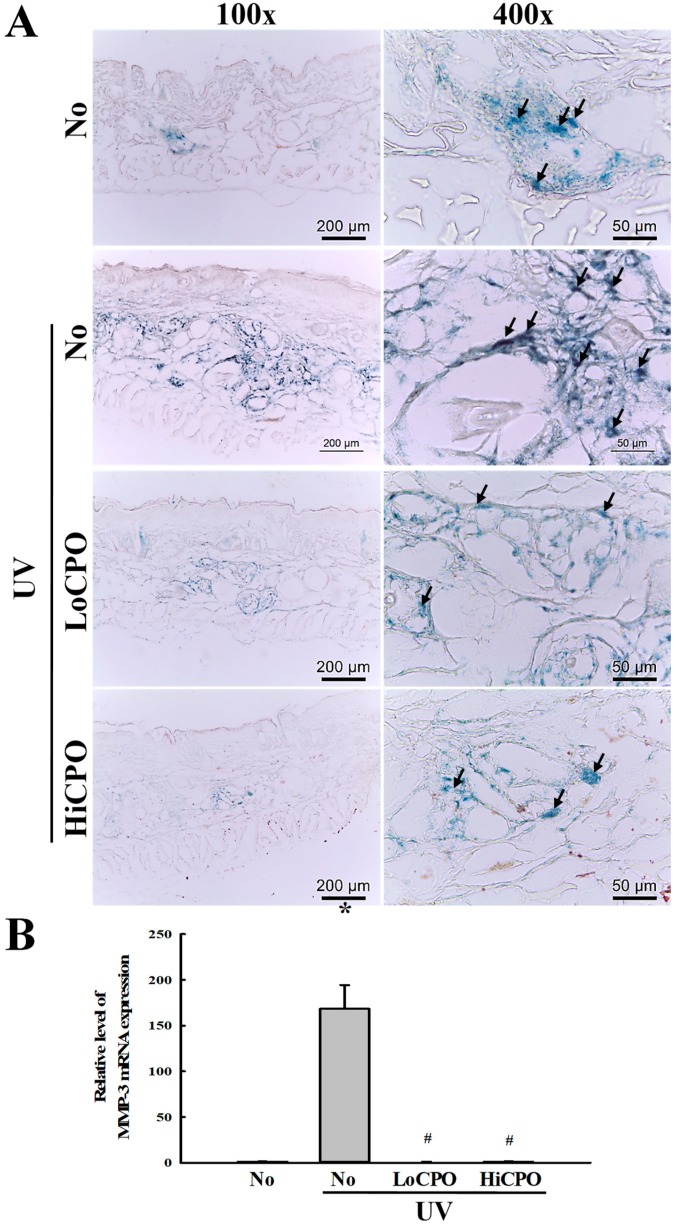
Level of galactosidase and MMP-3 mRNA. (**A**) Galactosidase staining analyses. The morphological features of cells stained with X-gal in dimethylformamide solvent at 37 °C, were observed in skin tissues at 400× magnification. Three to five mice per group were used and galactosidase levels were determined in duplicate for each mouse. (**B**) Expression level of MMP-3 mRNA. Levels of MMP3 in skin total mRNA were determined by quantitative real-time (qRT)-PCR using specific primers. mRNA levels were calculated with respect to β-actin mRNA (endogenous control). Three to five mice per group were used and PCR was conducted in duplicate. Data are reported as the means ± SD. * *p* < 0.05 compared to the No group. # *p* < 0.05 compared to the UV + No group. Abbreviations: LoCPO, low amount CPO; HiCPO, high amount CPO.

**Figure 10:**
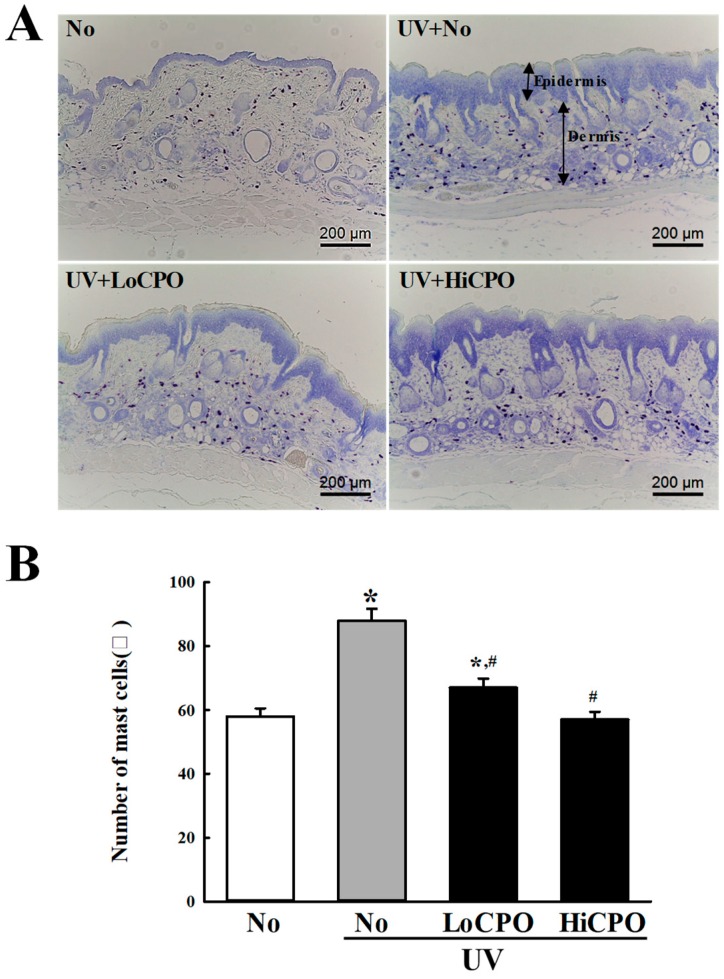
Number of mast cells into photoaging skin. Numbers of mast cells in skin tissue sections were determined by toluidine blue staining at 400× magnification. Total cells numbers in 1 mm^2^ were counted at three different sites per section. Three to five mice per group were used and mast cell numbers were determined in duplicate. Data are reported as the means ± SD. * *p* < 0.05 compared to the No group. # *p* < 0.05 compared to the UV + No group. Abbreviations: LoCPO, low amount CPO; HiCPO, high amount CPO.

Finally, we investigated the molecular mechanism responsible for the protective effects of CPO on UV-induced changes in skin aging biomarkers, by investigating the effect of CPO on UV-induced changes in galactosidase, MMP-3 mRNA, and mast cell numbers. To achieve these, the level of galactosidase was measured in frozen skin on the slide section after X-gal staining analysis, while MMP-3 mRNA was quantified in total skin RNA using quantitative real-time (qRT)-PCR method. UV exposure increased levels of galactosidase and MMP-3 mRNA, and both increases were inhibited by CPO (Figure 9). In addition, the distribution of mast cells was measured in dermis to investigate whether they completely reflect the level of MMP-3 mRNA. CPO also inhibited UV-induced increases in mast cell numbers (Figure 10). These observations suggest that the anti-photoaging effects of CPO may be associated with the suppressions of UV-induced increases in galactosidase level, MMP-3 mRNA expression and mast cell number.

## 3. Discussion

Oils derived from various plants are used as raw materials in the cosmetics industry and for medical purposes because they form a protective barrier on the skin [20]. The beneficial effects of almond, jojoba, soybean, and avocado oils have been confirmed in mammalian cells and in animal models [21]. Perilla oil has received considerable attention in the contexts the treatment of chronic diseases, such as cancer, diabetes, asthma, inflammatory, and cardiovascular diseases, because it contains fat, protein, vitamins, minerals, and phytochemicals [12]. As part of a research program aimed at identifying novel effects of perilla oil, we focused on the antiphotoaging effects of CPO on UV-treated skin fibroblasts and hairless mice. Our results provide novel scientific evidence that CPO treatment protects against UV-induced cellular damage. However, our results are limited because they were obtained based on observed changes in dorsal skin of a single animal model. Additional studies are thus required to clarify the protective role of CPO on photoaging and the molecular mechanism involved.

Plant oils contain many components including free fatty acids, triglycerides, glycerol, phenolic compounds, and tocopherols. In a previous study, five fatty acids, that is, linolenic acid, oleic acid, linoleic acid, palmitic acid, and stearic acid were detected in perilla oil at levels of 60.93%, 16.21%, 14.72%, 5.94%, and 2.20%, respectively [22]. These are similar to those found in the present study, though the amounts of linolenic and linoleic acid were slightly higher (64.11%) and lower (11.87%), respectively, in the present study. These differences are thought to have resulted from the process of oil extraction and raw materials.

The protective effects of UVB-induced damages were also examined using unsaponifiable matter (USM) from perilla seed meal [23]. Similar results detected in the present study were observed in USM-treated human skin fibroblasts (Hs68 cells) after UVB radiation although there are differences in the analysis factors. USM treatment at 1.0, 2.5 and 5.0 μg/mL suppressed UVB-induced cytotoxicity and ROS production in Hs68 cells [23]. Furthermore, decrease of total collagen production in UVB treated cells significantly recovered by USM treatment, while the opposite pattern observed on MMP1 and 3 secretion and mRNA under the same condition [23]. However, there is a difference in the composition between USM and CPO. USM contains high concentration of polycosanols, tocopherols, phytosterols and squalene [23].

Several studies have investigated the protective effects of plant oils on UV-induced cellular damages in mammalian cell lines. UV-induced DNA damage and apoptosis were significantly inhibited in NIH 3T3 cells by the essential oils of *C. officinale* and *L. chuanxiong* and were attributed to the downregulation of H2A.X. phosphorylation. In addition, these essential oil decreased the expression of p21, a key regulator of cell death, cell cycle arrest, and UVB-induced cell damages, while treatment with these oil inhibited the migration of UVB-damaged DNA and the expression of cyclin D1, which is involved in G1 to S phase transition [6]. In the present study, CPO significantly suppressed UV-induced changes in G2/M cell cycle arrest, ROS production, SOD activity, and MMP expression in NHDF cells. These results are similar to those obtained in a study on the protective effects of the essential oil from *C. officinale* and *L. chuanxiong* on UV-induced cycle arrest and damage [6]. However, some differences on the arrest phase of cell cycle have been observed between the present studies and previous one. These differences suggest that other regulatory factors, e.g., fatty acid type, influence cell cycle arrest. More studies are needed to improve understanding of the effects of plant oil components on cell cycle arrest.

Meanwhile, UV radiation are well known to be the main cause of skin photoaging in mammalian [24]. Especially, this response tends to be associated with UVA (320–340 nm wave length) and UVB (290–320 nm wave length) [25]. After exposure of UVA and UVB, the intracellular production of ROS and inflammatory mediators was excessively enhanced, and they were caused a triggering oxidative stress and unbalancing antioxidant defense system [26]. These alterations stimulate the structural and functional changes such as degradation of extracellular matrix (ECM) proteins and the upregulation of MMPs, thereby promoting skin photoaging [27]. In most previous studies, UVB irradiation onto the skin of hailress mice or keratinocytes was widely used to evaluate the therapeutic effects of natural products with anti-photogaing activity [28,29,30]. However, UVA and UVB expose induced a similar response on the MMP regulation of HDF cells although UVA radiation can be considered more suitable to study dermal fibroblast responses [28]. Based on above evidence, our study was selected UVB radiation to examine anti-photoaging effects and mechanism of CPO on the regulation of ECM in NHDF cells. Nevertheless, UVB radiation to induce photoaging in NHDF cells may be considered as a limiting factor in this study.

The number of mast cells increased around papillary dermis after UVB radiation although UVB rays cannot reach these area [31]. During this response, stem cell factor (SCF) lead to mast cells increase as well as neuropeptides from activated nerve ending promote mast cell degranulation [32,33]. Also, trypase secreted from mast cells during degranulation trigger the activation of MMPs and sequentially induce degradation of ECM proteins in dermis of skin [34]. In this study, total number of mast cells were counted in dermis of skin after UV + CPO treatment to examine whether alterations on the MMP level can be related with alteration on the distribution of mast cells. The decrease of mast cells was completely reflected MMP3 mRNA expression. Above results of the present study are in agreement with previous studies.

We also investigated the therapeutic effects of CPO on UV-induced photoaging. Topical application of CPO suppressed UV-induced changes in TEWL, skin hydration, and erythema and melanin indices and protected against histological changes. Plant oils contain different types and amounts of triglycerides, straight chain saturated fatty acids (SFAs) and unsaturated fatty acid (UFAs; e.g., linoleic, linolenic, and oleic acids) [20], and topical applications of SFAs and UFAs from different plants have been shown to significantly affect TEWL and irritant skin responses. In particular, treatments with oleic or arachidonic acid were reported to remarkably increase irritation response and TEWL, while SFAs caused slight irritation and increased TEWL [35]. Interestingly, linoleic acid plays an important role in maintaining skin barrier function to water permeability [36,37], whereas oleic acid tends to disrupt skin barrier functions and increase skin permeability to other compounds present in plant oils [38,39]

## 4. Materials and Methods

### 4.1. Subsection Preparation and Composition of CPO

Seed samples of *Perilla frutescens* were collected in October 2017 from plantations in Myrang City (Korea). Voucher specimens (WPC-18-001) were deposited in the Functional Materials Bank at the Pusan National University–Wellbeing RIS Center. Briefly, seeds of *P. frutescens* were washed with tap water and then dried in a hot-air drying machine (JSR, Seoul, Korea) for 24 h at 60 °C. CPO was produced using a cold pressing machine (Pungjin Food Machin Co., Mokpo, Korea) at 60 MPa and filtering through a 6–8 μm membrane filter (Figure 1A). The yield of CPO was calculated at 25–30 mL/100 g of seed sample. The compositions and calorie of CPO were analyzed in the Traditional Microorganism Resources Center of Keimyung University using an FID-equipped gas chromatograph (Agilent Technologies, Santa Clara, CA, USA). The calorie of CPO was measured at 829 Kcal/100 mL.

### 4.2. Cell Culture

NHDF cells were established from the dermis of juvenile foreskin or adult skin from different locations and were purchased from the ATCC (Manassas, VA, USA). Cells were grown in a humidified 5% CO_2_ and 95% atmosphere at 37 °C in Dulbecco’s Modified Eagle’s Medium (DMEM, Welgene, Gyeongsan-si, Korea) containing 10% fetal bovine serum, 2 mM glutamine, 100 U/mL of penicillin, and 100 μg/mL streptomycin.

### 4.3. Cell Viabilities

NHDF cell viabilities were determined using an MTT (3-[4,5-dimethylthiazol-2-yl]-2,5-diphenyltetrazolium bromide) assay (Sigma-Aldrich Co., St. Louis, MO, USA). Briefly, NHDF cells were seeded at a density of 3 × 10^4^ cells in 200 μL of DMEM, and cultured for 24–48 h at 37 °C incubator. When cells had attained 70–80% confluence, they were either treated with UVB (25, 50, 100, 200, or 400 mJ/cm^2^) and then CPO (0.5, 1, 1.5, 2, 2.5, or 3%). UVB irradiation was performed using a TL 20W/12 RS SLV/25 UVB Broadband TL lamp. Radiation intensities (mW/cm^2^) of UVB were measured at 30 cm from a light source using a UVP UVXTM Digital Radiometer (Analytik Jena US LLC, Upland, CA, USA). This value was converted to mJ/cm^2^ using the following formula; mJ = mW × S (second).

To determine the effect of CPO on UV-induced cellular damage, NHDF cells were divided into the following six groups; a UV + Vehicle (dH_2_O) group, a UV plus 50 μg/mL of gallotannin-enriched Galla rhois group (UV + GEGR group), a low concentration CPO group (0.625%; UV + LCPO group), a medium concentration CPO group (1.25%; UV + MCPO group), a high concentration CPO group (2.5%, UV + HCPO group), or a non-irradiated control group (No group). GEGR was used as positive control because it has high antioxidant activity [40]. CPO or GEGR were treated immediately after cells had been irradiated. After incubation for 24 h, supernatants were discarded, 2 mL of fresh DMEM and 50 μL of MTT solution (2 mg/mL in 1× PBS) were added per well, and cells were incubated at 37 °C for 4 h. The formazan precipitates formed were then dissolved in DMSO (dimethyl sulfoxide; Duchefa Biochemie, Haarlem, The Netherlands) and absorbance of each well was at 570 nm using a Vmax plate reader (Molecular Devices, Sunnyvale, CA, USA).

### 4.4. Intracellular ROS Analysis

Intracellular ROS levels were measured in NHDF cells by staining with 2′,7′-dichlorofluorescein diacetate (DCF-DA) (Sigma-Aldrich Co.) as previously described [41]. Briefly, NHDF cells seeded at 4.5 × 10^5^ cells/3 mL in 6-well plates were cultured to 70–80% confluent, irradiated at 50 mJ, and treated with CPO or GEGR and 25 μM DCFH-DA and incubated for 30 min at 37 °C. After washing twice with 1× PBS, the degree of green fluorescence was observed in DCF-DA stained cell under a fluorescence microscope (200×; Eclipse TX100, Nikon, Tokyo, Japan).

### 4.5. SOD Activity Analysis

SOD activity in NHDF cells was assessed using a calorimetric assay and an SOD assay kit (Dojindo Molecular Technologies Inc., Rockville, MD, USA). Briefly, NHDF cells were lysated with repetition of freezing and thawing in 100 μL of 1× PBS, and lysates were harvested by centrifugation at 5000× *g* for 5 min and stored at −70 °C until assayed. To determine SOD activity levels, lysates were diluted 1/1, 1/2, 1/2^2^, 1/2^3^, 1/2^4^, 1/2^5^, and 1/2^6^ with 1× PBS solution. Aliquots of sample solutions (20 μL) were then placed in individual wells of a 96-well plate along with 200 μL of WST-1 working solution and 20 μL of enzyme working solution and thoroughly mixed. The enzyme reaction was induced by incubation at 37 °C for 20 min, after which well absorbance was measured at 450 nm using a spectrophotometer. Finally, SOD activities were calculated using the following equation:

SOD activity (inhibition rate %) = [(A blank 1 − A blank 3) − (A sample − A blank 2)]/(A blank 1 − A blank 3) × 100(1)

where, A blank 1, 2, and 3 indicate the absorbance of blanks 1, 2, and 3, respectively, and ‘A sample’ is sample absorbance.

### 4.6. Cell Cycle Assay Using Flow Cytometry (FAM)

Cell cycle analysis was performed using a Muse™ Cell Cycle Kit (Millipore Co., Billerica, MA, USA) according to the manufacturer’s instructions. Briefly, NHDF cells were plated at 4.5 × 10^5^ cells/well and cultured in DME medium. Cells were treated with 50 mJ of UV and then with CPO or GEGR for 24 h, harvested by centrifugation at 3,000× *g* for 5 min, and fixed with 70% EtOH for 3 h at −20 °C. After washing with 1× PBS, cells were treated with Cell Cycle Reagent (200 μL) and incubated at 37 °C in a CO_2_ incubator for 30 min, number of cells in each phase of the cell cycle were analyzed by FAM (Millipore Co.).

### 4.7. Western Blot Analysis

The total proteins were obtained from NHDF cells and skin tissues using Pro-Prep Protein Extraction Solution (Intron Biotechnology Inc., Seongnam, Korea) based on the manufacturer’s protocol. After centrifugation at 13,000 rpm for 5 min, the protein concentrations were determined using a SMARTTM Bicinchoninic Acid Protein Assay Kit (Thermo Fisher Scientific Inc.). Proteins were separated by 4–20% SDS-PAGE (sodium dodecyl sulfate-polyacrylamide gel electrophoresis) for 2 h, and then transferred to nitrocellulose membranes at 40 V for 2 h. Membranes were then incubated at 4 °C with the following primary antibodies overnight; anti-MMP-1/8 (H-300) (Cat. No. SC-30069, Santa Cruz Biotechnology) or anti-β-actin antibody (Cat. No. A5316, Sigma-Aldrich Co.); washed with washing buffer (137 mM NaCl, 2.7 mM KCl, 10 mM Na2HPO4, and 0.05% Tween 20); and incubated with 1:1,000 diluted horseradish peroxidase (HRP)-conjugated goat anti-rabbit IgG (Cat. No. G21234, Invitrogen) at room temperature for 1 h. Blots were developed using Amersham ECL Select Western Blotting detection reagent (Cat. No. RPN2235, GE Healthcare, Little Chalfont, UK). Chemiluminescence signals from specific bands were detected using FluorChemi^®^FC2 (Alpha Innotech Co., San Leandro, CA, USA).

### 4.8. β-Galactosidase Staining

Staining degree of β-galactosidase in NHDF cells and skin tissues were measured using a β-Galactosidase Detection Kit (Cat No. 9860s, Cell Signal Co.) based on the manufacturer’s protocol. To detect an alteration on the β-galactosidase in NHDF cells, they were fixed with Fixative solution (1 mL) for 10–15 min at room temperature, washed with 1× PBS, treated with staining solution, and incubated at 37 °C overnight. Blue color development in cells was observed under a microscope (Leica Microsystems, Wetzlar, Germany) at 400× magnification.

To detect β-galactosidase in skin, tissues were embedded in OCT and slowly submerged into liquid nitrogen. Frozen tissue blocks were sectioned at 10 μm using a Leica Cryotome (Leica Microsystems). Tissue sections on slides were rinsed with distilled water and stained with the same kit as described above for NHDF cells. Color density in stained skin sections were observed under an optical microscope (Leica Microsystems).

### 4.9. Animal Studies

The Pusan National University–Institutional Animal Care and Use Committee reviewed and approved the animal protocol used in this study (Approval No. PNU-2018-0126). Mice were housed at the Pusan National University-Laboratory Animal Resources Center accredited by the Korean Food and Drug Administration (unit 000231) and the Association for Assessment and Accreditation of Laboratory Animal Care International (unit 001525). Male HR-1 hairless mice (six-weeks-old) were purchased from Central Lab Animal Inc. (Seoul, Korea). Water and a standard irradiated chow diet (Samtako BioKorea Co., Osan, Korea) were provided ad libitum to all animals throughout the experimental period. Furthermore, all mice were maintained under specific pathogen-free conditions (50 ± 10% RH/23 ± 2 °C) under a strict light:dark cycle (lights on at 08:00 h and off at 20:00 h).

Mice (*n* = 21) were assigned to a non-treated control group (No group, *n* = 7), a UV + No group, a UV + low amount CPO group (UV + LoCPO group, *n* = 7) or a UV + high amount CPO group (UV + HiCPO group, *n* = 7). Mice in the two CPO treated groups were treated topically with 50 uL or 100 uL of CPO onto dorsal skin three times a week for 4 weeks after being irradiated with UVB. To determine the 1 minimal erythema dose (MED), the dorsal skin of the mice was exposed to different doses of UVB light and the formation of erythema was evaluated after 24 h. Skin photoaging was then induced by irradiation at 1 MED three times/week (Monday, Wednesday and Friday) for first week. After that week, the dose of UV radiation was gradually increased by 1 MED per week from 1 to 4 MED and treated with in the same way.

Wrinkle formation, hydration, erythema index, TEWL and melanin indices were measured at 24 h after the 4-week treatment period in dorsal skins of mice anesthetized using Alfaxan (Jurox, Kansas, USA; i.p., 80 mg/kg body weight) [42]. After conducting these assessments, mice were promptly sacrificed using CO_2_ to acquire skin tissue samples.

### 4.10. Evaluation of Wrinkle Formation

Wrinkle formation was measured by the procedure established by our laboratory using a DETAX System II (MIXPAC) and Double-Stick Disc (3M Health Care, Neuss, Germany) [43]. After final treatment, skin surface impressions were prepared by applying liquid silicon rubber delivered using the DETAX System II to dorsal skins. Each replica was captured using a digital camera connected to a Leica EZ4HD (Leica Microsystems). Depths and numbers were recorded and samples were then as described by Bissett et al. [19], where Grade 0 = no wrinkle formation, Grade 1 = some shallow wrinkles, Grade 2 = obvious wrinkles, and Grade 3 = several deep wrinkles.

### 4.11. Skin Phenotypes

Dorsal skins of all mice were assessed for TEWL, erythema and melanin severities, and hydration [43]. TEWLs were measured using a Corneometer TM300 (Courage and Khazaka Electronics, Cologne, Germany), erythema and melanin indices were determined using a Mexameter MX18 (Courage and Khazaka Electronics, Cologne, Germany), and hydration was measured using a Corneometer CM825 (Courage and Khazaka Electronics, Cologne, Germany). Measurements were performed in duplicate at three dorsal skin sites per mouse.

### 4.12. Histopathological Analysis

Mouse skin tissues were fixed overnight in 10% neutral buffered formaldehyde (pH 6.8), dehydrated using an alcohol series, and embedded in paraffin wax. Sections (4 μm) were cut using a Leica microtome (Leica Microsystems), deparaffinized with xylene (DaeJung, Gyeonggi-do, Korea), rehydrated using graded ethanol (100 to 70%), washed with distilled water, stained with hematoxylin and eosin (H&E, #HT110332, Sigma-Aldrich Co.), and washed with dH_2_O. Pathological changes were assessed using the Leica Application Suite (Leica Microsystems).

Number of mast cells in skin were determined by toluidine blue staining, as previously described [44]. Briefly, after deparaffinization and rehydration, skin sections on slides were stained with 0.25% toluidine blue (Sigma-Aldrich Co.), and then examined under an optical microscope for the presence of mast cells. Numbers of mast cells per mm^2^ were determined using the Leica Application Suite (Leica Microsystems).

### 4.13. Quantitative Real Time-PCR Analysis

Frozen skin tissues were chopped with a scissors and homogenized in RNA Bee solution (#CS-105B, Tet-Test Inc., Friendswood, TX, USA). Total RNA was isolated by centrifugation at 15,000 rpm for 15 min, and total RNA concentrations were determined by UV spectroscopy. The complementary DNA (cDNA) was synthesized using Invitrogen Superscript II reverse transcriptase (Thermo Scientific, Wilmington, DE, USA), and quantitative PCR was performed using the cDNA template (1 μL) and 2× Power SYBR Green (6 μL; Toyobo Life Science, Osaka, Japan) containing specific primers. The primer sequences used for target gene expression identification were as follows: MMP-3, sense 5’- GTT GGT GGC TTC AGT ACC TT -3’ and anti-sense, 5’- GAT GAA CGA TGG ACA GAG GA -3’; β-actin, sense 5′- TGG AAT CCT GTG GCA TCC ATG AAA C -3′ and anti-sense 5′- TAA AAC GCA GCT CAG TAA CAG TCC G -3′. qPCR was performed over 40 cycles of denaturation at 95 °C for 15 s, annealing 70 °C for 60 s, and extension 70 °C for 60 s. Fluorescence intensities were measured at the end of the extension phase of each cycle. Threshold values for sample fluorescence intensities were set manually, and reaction cycles during which PCR products exceeded this fluorescence intensity thresholds during the exponential phase were considered as threshold cycles (Cq). Expressions of MMP3 gene were quantified with respect β-actin (the housekeeping gene) by comparing Cq values at constant fluorescence intensity, as described by Livak and Schmittgen [45].

### 4.14. Statistical Significance Analysis

Statistical analyses were performed using SPSS release 10.10 for Windows (IBM SPSS, SPSS Inc., Chicago, IL, USA). The significances of intergroup differences were determined by one-way analysis of variance followed by Tukey’s post hoc test for multiple comparisons. Results are presented as means ± SDs, and statistical significance was accepted for *p* values < 0.05.

## 5. Conclusions

The present study demonstrates that CPO ameliorates UV-induced cellular damage and photoaging in NHDF cells and HR-1 hairless mice, respectively, and indicates these effects are mediated by the effects of CPO on antioxidant activity, cell cycle regulation and ECM regulation. These observed regulatory effects of CPO on UV-induced cellular damage and photoaging suggest CPO be viewed as a potential anti-photoaging drug that protects skin and stimulates skin repair.
